# Efficacy and safety of nicoboxil/nonivamide ointment for the treatment of acute pain in the low back – A randomized, controlled trial

**DOI:** 10.1002/ejp.719

**Published:** 2015-04-30

**Authors:** M. Gaubitz, T. Schiffer, C. Holm, E. Richter, W. Pisternick‐Ruf, T. Weiser

**Affiliations:** ^1^ Interdisciplinary Diagnostics and Therapy in the Academy for Manual MedicineUniversity MünsterGermany; ^2^ Outpatient Clinic for Sports Traumatology and Public Health ConsultationGerman Sport University CologneGermany; ^3^ Boehringer Ingelheim RCV GmbH & Co KGViennaAustria; ^4^ Boehringer Ingelheim Pharma GmbH & Co. KGIngelheimGermany; ^5^ Boehringer Ingelheim Pharma GmbH & Co. KGBiberachGermany

## Abstract

**Background:**

Until now, nonivamide/nicoboxil ointment has not been tested in a randomized trial for the treatment of acute non‐specific low back pain.

**Methods:**

This phase III randomized, double‐blind, active‐ and placebo‐controlled, multi‐centre trial investigated efficacy, safety and tolerability of topical nicoboxil 2.5%/nonivamide 0.4% for treatment of acute non‐specific low back pain [primary endpoint: pain intensity (PI) difference between pre‐dose baseline and 8 h after the first application].

**Results:**

Patients (*n* = 805), 18–74 years of age were treated for up to 4 days with nicoboxil 2.5%/nonivamide 0.4%, nicoboxil 2.5%, nonivamide 0.4% or placebo ointment. Pre‐dose baseline pain intensity (6.6 on a 0‐ to 10‐point numerical rating scale) was reduced by 1.049 points with placebo, by 1.428 points with nicoboxil, by 2.252 points with nonivamide and by 2.410 points with nicoboxil/nonivamide after 8 h (*p* < 0.0001 for nicoboxil/nonivamide vs. placebo, nicoboxil; *p* = 0.4171 for nicoboxil/nonivamide vs. nonivamide). At the end of treatment, the combination provided more pronounced PI reduction (3.540 points) compared with nicoboxil (2.371, *p* < 0.0001), nonivamide (3.074, *p* = 0.0259) and placebo (1.884, *p* < 0.0001). Low back mobility scores on Day 1 were better for the combination compared with all other treatments (*p* < 0.044); on Day 2–4, scores were better than for placebo and nicoboxil (*p* < 0.003). Patients assessed efficacy of the combination as greater than of the comparators (*p* ≤ 0.0129). All treatments were tolerated well. No treatment‐related serious adverse events were reported.

**Conclusion:**

Nicoboxil/nonivamide ointment is an effective, well‐tolerated medication for the treatment of acute non‐specific low back pain.

**What's already known about this topic?**

- Low back pain is a major health and socio‐economic problem.
- Clinically proven and effective treatment options are sparse.

**What does this study add?**

- Nonivamide/nicoboxil ointment is an effective, well‐tolerated medication for the treatment of acute non‐specific low back pain.

## 1 Introduction

Low back pain is a major health and socio‐economic problem in many countries. Point prevalence of up to 58%, and 1‐year prevalence of up to 82% are reported (Hoy et al., 2010). In most cases, specific causes for low back pain cannot be identified (i.e. non‐specific low back pain). Usually, low back pain is a self‐limiting disorder, and approximately 90% of patients experience remission within 6 weeks (Casser, 2012). Nevertheless, low back pain causes a high burden on the affected patients and accounts for high direct costs associated with clinical diagnostics and therapy, as well as high indirect costs due to the inability to work (German Medical Association (BÄK), 2013).

Despite the high burden of (acute) non‐specific low back pain, clinically proven treatment options are relatively sparse. Early intervention is desirable to get patients back to their normal activities as soon as possible and to prevent chronification. Regarding pharmacotherapy, paracetamol is usually recommended as first‐line therapy and NSAIDs (non‐steroidal anti‐inflammatory drugs) as second‐line therapy when paracetamol is not sufficient. In addition, opioids, muscle relaxants, steroids, antidepressants or anticonvulsive medication are recommended by some guidelines (Koes et al., 2010). Overall, clinical data for low back pain treatment with these medications are limited, and there is still need for new treatment options. Moreover, the use of systemic analgesics like paracetamol and NSAIDs may be limited based on potential side effects (e.g. gastrointestinal complications and kidney failure in the elderly) and other risk factors (e.g. known drug interactions). Topical treatments are considered to have a better side effect profile compared with systemic medication.

Topical nicoboxil/nonivamide ointment (Finalgon^®^, Boehringer Ingelheim, Germany) has been used since the 1950s to treat discomfort of the musculoskeletal system (Ward, 1955; Chitil and Ortner, 1959). The nicotinic acid ester nicoboxil has vasodilatory properties (‘rubefacient’). Nonivamide is structurally closely related to capsaicin and has very similar effects at the target receptor TRPV1 (Weiser et al., 2012). Rubifacients, as well as topical capsaicin have analgesic effects (Higashi et al., 2010; Bley, 2013). Moreover, topical nicoboxil/nonivamide has been shown to induce hyperaemia in skin and in the musculature below the treated skin area (Warnecke et al., 2013), which can be anticipated to have beneficial effects upon muscular complaints.

Despite its long availability, the effects of nicoboxil/nonivamide ointment for the treatment of acute low back pain have not been investigated in a randomized controlled trial.

This randomized, double‐blind, placebo‐controlled, multi‐centre study examined the efficacy, tolerability and safety of nicoboxil 2.5%/nonivamide 0.4% ointment in the treatment of adults with acute low back pain.

## 2 Methods

### 2.1 Study design

This was a phase III, multi‐centre, randomized, active‐ and placebo‐controlled, double‐blind, parallel‐group, four‐arm study in patients 18–74 years of age with acute non‐specific low back pain. The study was performed at 37 sites in Germany (mainly general practitioners) between October 16, 2012 and April 19, 2013. The primary objective was to demonstrate superior efficacy of an ointment containing 2.5% nicoboxil and 0.4% nonivamide over placebo and ointments containing 2.5% nicoboxil alone and 0.4% nonivamide alone for the treatment of acute low back pain.

### 2.2 Ethical considerations

Prior to start of the study, the clinical trial protocol, the Patient Information leaflet, the Informed Consent Form and other locally required documents were reviewed by the Independent Ethics Committees and/or Institutional Review Boards of the participating centres and the competent authority (Bundesinstitut für Arzneimittel und Medizinprodukte, Bonn, Germany).

The trial was carried out in compliance with the clinical trial protocol, the principles of the Declaration of Helsinki, the ICH‐GCP, and with applicable regulatory requirements and Boehringer Ingelheim standard operating procedures. Prior to enrolment, patients gave their written informed consent according to German Drug Law and the ICH‐GCP.

### 2.3 Patient population

Adults 18–74 years of age with acute low back pain for more than 2 days and less than 21 days, and a low back pain rating ≥5 on an 11‐point (0–10) numerical rating scale (NRS) were enrolled. Although the trial protocol stipulated the inclusion of patients up to 65 years of age, a number of older patients were also enrolled and not excluded from the analyses. Women of childbearing potential had to have a negative urine pregnancy test and be using a highly effective method of birth control.

Patients were to be excluded for the following reasons: Multilocular pain or panalgesia; history of more than three low back pain episodes in the previous 6 months; patients with low back pain due to neurological causes [abnormal findings in at least one of the following assessments: achilles tendon reflex, patella reflex, heel walking, toe walking, cutaneous sensitivity of the legs (like hypo‐ or hyperaesthesia, allodynia, hyperalgesia) including gluteal region], paresis tests in supine position upon dorsiflexion, plantar flexion, hip flexion, knee extension, bladder and/or rectum dysfunction; acute low back pain due to vertebral collapse or neoplastic, inflammatory (ankylosing spondylitis), traumatic or infective origins; any condition, disease or concomitant treatment that in the judgement of the investigator affected the patient's ability to participate in the clinical trial or that influenced the test methodology used; negative experience in the past with heat treatment for muscle complaints; history of treatment of back pain with centrally acting analgesics (e.g. opioids) and muscle relaxants; surgery due to back pain or rehabilitation due to back pain in the previous 12 months; spinal injection back pain treatment within 6 months prior to enrolment; intake of antidepressant/antipsychotic medication within 4 weeks prior to enrolment; treatment of the recent low back pain period with oral analgesics for more than four consecutive days; locally applied medication to the back within 48 h prior to enrolment; administration of other analgesics within 24 h prior to enrolment (exception: acetylsalicylic acid up to 100 mg/daily for anti‐platelet aggregation therapy); non‐pharmacological low back pain treatment [physiotherapy, heat treatment (e.g. hot water bottle, heat patch) or massages] within 12 h prior to enrolment; participation in an investigational drug or device trial within 4 weeks prior to enrolment; hypersensitivity to nicoboxil, nonivamide or paracetamol; known hypersensitivity to any other ingredient; skin lesions (e.g. rash, dermatitis, bruising, laceration) in the back region; drug dependence and/or alcohol abuse; severe hepatocellular insufficiency; patients who were pregnant or breastfeeding.

### 2.4 Treatments

Patients were randomly assigned to one of the four treatment groups (nicoboxil/nonivamide, nicoboxil, nonivamide and placebo) in a 1:1:1:1 ratio at Visit 1. The randomization list was generated using a validated system using a pseudo‐random number generator so that the resulting treatment was both reproducible and non‐predictable. A random block size of 4 was used. Access to the treatment codes was controlled and documented.

Study medication for each patient was packaged in a box containing two tubes of ointment. Patients had to administer one of the four ointments up to three times a day for a maximum of 4 days. The first 2 doses were to be applied at baseline (time point 0 at Day 1) and after 4 h on Day 1. After the second application on Day 1, patients could administer study medication on a PRN (as needed) basis but not earlier than 8 h after the first application for up to a total of 4 days. The intervals between each application of study medication had to be at least 4 h; a maximum of three applications in a 24‐h period was allowed.

Two centimetres of ointment was applied to an area of about 20 cm × 20 cm on the patient's low back, where he/she felt the pain to be most pronounced. The whole application was to be completed within 3 min.

Paracetamol tablets were allowed as rescue medication (up to a maximum of three times 1 g per day).

### 2.5 Efficacy endpoints

Patients were to assess the intensity of their low back pain on an 11‐point NRS ranging from 0 = ‘no pain’ to 10 = ‘worst pain possible’ in a diary. Time points of assessment were pre‐dose (i.e. directly before the first application of study drug at Visit 1) and 0.5, 1, 2, 3, 4, 6, and 8 h after the first ointment application on Day 1. The pre‐dose pain intensity score was used as baseline PI. Furthermore, patients assessed the average pain intensity (API) of their low back pain on the same rating scale in the diary at the end of each treatment day. Primary endpoint was pain intensity difference (PID) between baseline (pre‐dose) and 8 h after the first application (PID_8h_). Secondary endpoints were PID between baseline (pre‐dose) and 4 h after the first application (PID_4h_), average PID versus baseline on the last individual treatment day (APID_LID_), and patient assessment of efficacy on the last individual treatment day [on a 4‐point verbal rating scale (VRS) with the categories, ‘poor’, ‘fair’, ‘good’, ‘very good’].

Other efficacy endpoints included assessment of APID from baseline to Day 4, time to onset of pain relief (7‐point VRS with categories ‘within 30 min’, ‘between 30 min and 1 h’, ‘between 1 and 2 h’, ‘between 2 and 4 h’, ‘between 4 and 8 h’, ‘after more than 8 h’, ‘no effect’), mobility score (Question: ‘How did the treatment affect your low back mobility today?’, 4‐point VRS with categories ‘no or poor improvement’, ‘fair improvement’, ‘good improvement’, ‘very good improvement’) and number of patients taking rescue medication.

### 2.6 Safety endpoints

Adverse events (AEs) and serious adverse events (SAEs) were defined according to the International Conference for Harmonization Note for Guidance on Clinical Safety Data Management: Definitions and Standards for Expedited Reporting. Changes in vital signs and physical examination results were recorded as AEs or SAEs if they were judged clinically relevant by the investigator. Investigators recorded the intensity of AEs as mild (signs or symptoms that were easily tolerated), moderate (enough discomfort to cause interference with usual activity) or severe (incapacitating or causing inability to work or to perform usual activities). Investigators recorded the relatedness as possibly related or as not related to the study treatment.

Tolerability was assessed by the investigators at Visit 2 (end of study) using a 4‐point VRS (0 = ‘poor’, 1 = ‘fair’, 2 = ‘good’, 3 = ‘very good’).

### 2.7 Sample size

For the primary endpoint, PID at 8 h, a difference of 1 point on the 11‐point NRS (0–10) between the treatment groups, together with a common standard deviation of 3 was used to calculate the sample size. With a sample size of 192 per group (768 total), a power of 90% was provided assuming a two‐sided *t*‐test with α = 0.05.

### 2.8 Statistical analysis

#### 2.8.1 Definition of data sets

The ‘treated set’ (TS) was defined as all randomized patients who used at least one dose of study medication. The ‘full analysis set’ (FAS) included all patients of the TS who provided any post‐treatment data for the primary efficacy endpoint. The ‘per‐protocol set’ (PPS) was composed of all patients who were part of the FAS and complied with the protocol without any important protocol violations (IPVs). Efficacy endpoints were to be assessed in the FAS; safety endpoints in the TS.

#### 2.8.2 Primary endpoint

For the primary endpoint, PID at 8 h, the analysis used a restricted maximum likelihood‐based repeated measures approach and included all available longitudinal pain intensity observations at each post‐baseline time point up to 8 h. The statistical model was applied to the analysis of change from baseline in pain intensity at 0.5, 1, 2, 3, 4, 6 and 8 h, and included the fixed, categorical effects of centre, treatment, time, and treatment‐by‐time interaction, the continuous covariate of baseline pain intensity and the residual error term. Centres with less than four evaluable patients for the primary analysis were pooled together with larger centres (this was the case for two centres). Within‐patient errors were modelled by unstructured covariance. The Kenward–Roger approximation was used to estimate the denominator degrees of freedom. Differences between the treatment group effects with regard to the primary endpoint of PID at 8 h were estimated by reference to the adjusted least square means and the corresponding 95% confidence intervals (CIs). Sensitivity analysis was performed on the PPS (i.e. all patients of the FAS who did not have any IPVs that would have affected the primary endpoint).

#### 2.8.3 Secondary and other analyses

PID at 4 h and APID on Day 1 to Day 4 were analysed with the corresponding model used for the primary endpoint. APID_LID_ was analysed by analysis of covariance, with treatment and centre as fixed effects and baseline pain intensity as a continuous covariate. Patient assessment of efficacy on the last individual treatment day was analysed by a cumulative logit model fit to the underlying ordinal data with treatment and centre as fixed effects and baseline pain intensity as a continuous covariate. Adjusted odds ratios together with 95% CIs were used to quantify the effect of treatment and to compare the treatment groups. For the endpoint ‘time to onset of pain relief’, a Kaplan–Meier analysis was performed, and the log rank test stratifying for the variable baseline PI was used to determine the differences between the treatment groups. The time was censored at 9 h post‐dose and displayed as >8 h. The mobility score on each treatment day was analysed corresponding to the assessment of efficacy on the LID, but adding the fixed factors day, and treatment‐by‐day interaction. Safety evaluation was performed on the TS (i.e. all randomized patients who used at least one dose of study medication).

#### 2.8.4 General considerations

Missing baseline pain intensity values were replaced by the next available post‐dose value if post‐treatment data were available within the first hour. A patient's daily overall assessment of efficacy was assigned ‘poor’ if it was missing because of discontinuation due to lack of efficacy; it was assigned ‘very good’ if the patient discontinued after the second application and had a pain intensity of 0 at 8 h post‐dose. For the primary analysis (FAS), the number of applications was recorded as 1 if missing on Day 1 in the diary, because all patients performed the first ointment application at the site. However, such patients were excluded from the PPS. No imputations or replacements were made for analyses utilizing a likelihood‐based repeated measures model as well as for other missing data.

All statistical testing was performed using SAS^®^ version 9.2 (SAS Institute, Cary, NC, USA) and with a two‐sided alpha of 0.05.

## 3 Results

### 3.1 Patients

A total of 805 patients were enrolled and randomized; 202 were randomized to nicoboxil/nonivamide, 198 to nonivamide alone, 201 to nicoboxil alone and 204 to placebo (Full analysis set; Supporting Information Figure S1). All patients were treated. Sex, age, race, baseline back pain and duration of back pain were similar in the four treatment groups (Table 1). Before patients received trial medication, intensity of their low back pain was 6.6 ± 1.13 points on the 11‐point NRS with duration of 6.4 ± 3.91 (means ± SD) days. Medical history was similar in the treatment groups (data not shown). A total of 578 patients completed the trial. Of the 805 randomized patients, 227 (28.2%) prematurely discontinued the trial medication; this included 86 in the placebo group, 62 in the nicoboxil group, 46 in the nonivamide group and 33 in the nicoboxil/nonivamide group. The main reason for premature discontinuations (*n* = 194) was lack of efficacy. This was most frequent in patients treated with placebo (42.2%), accounting for 84 of 86 discontinuations in this treatment group. By contrast, premature discontinuation owing to lack of efficacy was reported for 15 (7.4%) patients treated with nicoboxil/nonivamide, 35 (17.7%) treated with nonivamide alone and 60 (29.9%) treated with nicoboxil alone. AEs led to premature discontinuation of treatment in 13 patients (6.4%) in the nicoboxil/nonivamide group, 9 (4.5%) in the nonivamide group and 1 (0.5%) in the placebo group. None of these AEs was considered serious. Ten additional patients prematurely terminated the study for the reasons ‘non‐compliant with protocol’ (one patient under nicoboxil/nonivamide), ‘refused to continue trial medication’ (one patient under nicoboxil, two under nonivamide, two under nicoboxil/nonivamide), ‘other’ (one patient under placebo, one under nicoboxil, two under nicoboxil/nonivamide).

**Table 1:** Patient baseline demographics and baseline characteristics

	Placebo	Nicoboxil	Nonivamide	Nixoboxil/Nonivamide	Total
Number of patients, *n* (%)	204 (100.0)	201 (100.0)	198 (100.0)	202 (100.0)	805 (100.0)
Gender, *n* (%)					
Male	110 (53.9)	110 (54.7)	95 (48.0)	102 (50.5)	417 (51.8)
Female	94 (46.1)	91 (45.3)	103 (52.0)	100 (49.5)	388 (48.2)
Race, *n* (%)					
White	202 (99.0)	199 (99.0)	195 (98.5)	200 (99.0)	796 (98.9)
Black/African American	2 (1.0)	2 (1.0)	3 (1.5)	1 (0.5)	8 (1.0)
American Indian/Alaska Native	0 (0.0)	0 (0.0)	0 (0.0)	1 (0.5)	1 (0.1)
Ethnicity, *n* (%)					
Not Hispanic/Latino	202 (99.0)	199 (99.0)	198 (100.0)	200 (99.0)	799 (99.3)
Hispanic/Latino	2 (1.0)	2 (1.0)	0 (0.0)	2 (1.0)	6 (0.7)
Age, mean (SD), years	39.2 (13.27)	40.4 (13.06)	42.2 (13.53)	40.2 (14.31)	40.5 (13.57)
Height, mean (SD), cm	173.1 (9.79)	173.1 (9.21)	171.6 (9.66)	172.8 (9.57)	172.7 (9.56)
Weight, mean (SD), kg	78.44 (17.558)	80.07 (17.297)	78.16 (15.135)	80.97 (17.113)	79.41 (16.819)
BMI, mean (SD), kg/m^2^	26.09 (5.127)	26.68 (5.370)	26.54 (4.826)	27.08 (5.300)	26.59 (5.164)
Baseline pain intensity, mean (SD)	6.6 (1.12)	6.5 (1.14)	6.7 (1.12)	6.7 (1.13)	6.6 (1.13)
Duration of pain, mean (SD), days	6.3 (3.56)	6.6 (3.97)	6.2 (3.96)	6.7 (4.12)	6.4 (3.91)

### 3.2 Compliance

Ointment was applied only once (instead of twice) on Day 1 by one patient (0.5%) treated with nicoboxil/nonivamide, four (2.0%) treated with nonivamide alone, one (0.5%) treated with nicoboxil and four (2.0%) treated with placebo. Additionally, two patients in the placebo group applied ointment four times on several treatment days, even though the allowed maximum number of applications was three. All other patients complied with the treatment instructions. The mean weight of ointment per application was similar for the four treatment groups (data not shown).

### 3.3 Efficacy

#### 3.3.1 Primary endpoint: PID at 8 h

**Figure 1:**
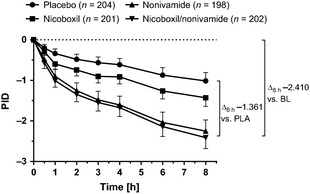
Pain intensity differences (PIDs) up to 8 h after the first application of ointment containing placebo (PLA), nicoboxil, nonivamide or the combination of nicoboxil and nonivamide. Ointment was applied at 0 and 4 h. The combination reduced PID by more than 1 point compared with placebo, and by more than 2 points compared with baseline (BL). Data are shown as adjusted means with 95% confidence intervals (CI). The statistical model included baseline pain intensity, centre, time (0.5 h, 1 h, 2 h, 3 h, 4 h, 6 h, 8 h), treatment and treatment‐by‐time interaction.

At 8 h, pain intensity decreased statistically significantly more in the nicoboxil/nonivamide group (adjusted mean PID [95% CI] = −2.410 [−2.683, −2.138]) than in the nicoboxil group (−1.428 [−1.642, −1.213]; *p* < 0.0001) and in the placebo group (−1.049 [−1.257, −0.841]; *p* < 0.0001) (Fig. 1, Table 2). The PID at 8 h was numerically but not statistically significantly different between the nonivamide group (−2.252 [−2.529, −1.975]; *p* = 0.4171) and the nicoboxil/nonivamide group.

**Table 2:** Adjusted mean PID between baseline and 8 h after the first application and adjusted mean difference between PI at baseline and API on the last individual treatment day

	*N* analysed	Mean baseline PI	Adjusted^a^ mean PID ± SE (95% CI)	Adjusted^a^ mean difference vs. Nicoboxil/Nonivamide ± SE (95% CI) and *p*‐value
Adjusted mean PID between baseline and 8 h after the first application (FAS)				
Nicoboxil/Nonivamide	202	6.703	−2.410 ± 0.138 (−2.683, −2.138)	
Placebo	204	6.613	−1.049 ± 0.106 (−1.257, −0.841)	−1.362 ± 0.171 (−1.699, −1.025) <0.0001
Nicoboxil	201	6.517	−1.428 ± 0.109 (−1.642, −1.213)	−0.983 ± 0.173 (−1.324, −0.642) <0.0001
Nonivamide	198	6.672	−2.252 ± 0.141 (−2.529, −1.975)	−0.158 ± 0.195 (−0.541, 0.225) 0.4171
	*N* analysed^b^	Mean baseline PI	Adjusted^c^ mean PID ± SE (95% CI)	Adjusted^a^ mean difference vs. Nicoboxil/Nonivamide ± SE (95% CI) and *p*‐value
Adjusted mean difference between PI at baseline and API on the last individual treatment day (all analysed patients of the FAS)				
Nicoboxil/Nonivamide	199	6.693	−3.540 ± 0.159 (−3.853, −3.226)	
Placebo	200	6.625	−1.884 ± 0.160 (−2.198, −1.570)	−1.655 ± 0.208 (−2.064, −1.247) <0.0001
Nicoboxil	200	6.520	−2.371 ± 0.160 (−2.685, −2.057)	−1.169 ± 0.209 (−1.578, −0.759) <0.0001
Nonivamide	198	6.672	−3.074 ± 0.161 (−3.390, −2.758)	−0.466 ± 0.209 (−0.875, −0.056) 0.0259

a The statistical model included baseline PI, centre, time, treatment and treatment‐by‐time interaction.

b Patients with measurements available on the last individual treatment day; therefore, baseline values slightly differ from those for PID_8h_.

c The statistical model included baseline PI, centre and treatment.

Compared with placebo, the combination reduced pain intensity 8 h after the onset of treatment by 1.362 points more.

Sensitivity analyses of the primary endpoint using the PPS and subgroup analyses by gender confirmed the results of the primary analyses (data not shown).

#### 3.3.2 Secondary and other endpoints

Four hours after the onset of treatment, pain intensity decreased significantly more in the nicoboxil/nonivamide group (adjusted mean PID [95% CI] = −1.699 [−1.914, −1.484]) than in the nicoboxil group (−0.968 [−1.147, −0.789]); *p* < 0.0001) and in the placebo group (−0.650 [−0.802, −0.497]; *p* < 0.0001). The PID at 4 h was not significantly different between the nonivamide (−1.641 [−1.858, −1.424]; *p* = 0.7037) and the nicoboxil/nonivamide groups.

Compared with placebo, pain intensity was reduced by 1.049 points more in the combination group at this early time point.

At the last individual treatment day, API decreased from baseline more in the nicoboxil/nonivamide group compared with the three other treatments (*p* < 0.0001 vs. placebo and nicoboxil, and *p* = 0.0259 vs. nonivamide) (Table 2).

**Figure 2:**
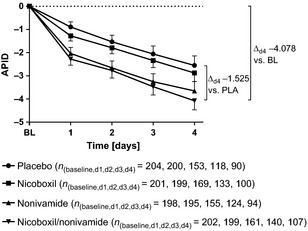
Average pain intensity differences (APIDs) from Day 1 to Day 4. All treatments reduced APID over the treatment period, with the most pronounced effects for nicoboxil/nonivamide. The number of patients with available PI data for the treatment days shows that a relatively high number did not continue treatment over the entire (allowed) 4‐day period. Data are shown as adjusted means with 95% confidence intervals (CI). The statistical model included baseline pain intensity, centre, time (Day 1, Day 2, Day 3, Day 4), treatment and treatment‐by‐time interaction.

The adjusted mean APID continuously decreased from Day 1 to Day 4 in all treatment groups and the absolute value of the APID was on all treatment days numerically highest in the nicoboxil/nonivamide group, followed by the nonivamide group, the nicoboxil group and the placebo group (Fig. 2). On all treatment days, the treatment differences based on the adjusted mean APID were in favour of nicoboxil/nonivamide compared with nicoboxil alone (*p* < 0.0001), placebo (*p* < 0.0001) and nonivamide (*p* ≥ 0.1324). Table 3 summarizes the PID values for primary, secondary and other pain‐related endpoints and treatments.

**Table 3:** Pain intensity differences compared with baseline for the various pain‐related endpoints. Percentages for change from baseline are rounded

	Placebo	Nicoboxil	Nonivamide	Nicoboxil/nonivamide
	PI score, mean (%)	PI score, mean (%)	PI score, mean (%)	PI score, mean (%)
Baseline PI^a^, mean	6.613 (100)	6.517 (100)	6.672 (100)	6.703 (100)
Change from baseline				
PID_4h_. adjusted mean	−0.650 (−10)	−0.968 (−15)	−1.641 (−25)	−1.699 (−25)
PID_8h_. adjusted mean	−1.049 (−16)	−1.428 (−22)	−2.252 (−34)	−2.410 (−36)
APID_LID_. adjusted mean	−1.884 (−28)	−2.371 (−36)	−3.074 (−46)	−3.540 (−53)
APID_d4_, adjusted mean	−2.553 (−39)	−2.876 (−44)	−3.654 (−55)	−4.078 (−61)

a Full Analysis Set.

Time to onset of pain relief was assessed using a 7‐point VRS (using a Kaplan–Meier analysis). Based on the distribution of patient frequencies in the different categories of time to onset of pain relief within the first 8 h, the combination of nicoboxil and nonivamide had an earlier onset of pain relief than the other three treatment groups. The median category of time to onset of pain relief was lowest for the combination of nicoboxil and nonivamide (between 1 and 2 h), followed by the nonivamide group (between 2 and 4 h), and the nicoboxil group (between 4 and 8 h), and it was highest for placebo (after more than 8 h; Supporting Information Table S1).

In the nicoboxil/nonivamide group, patients assessed the improvement of their mobility as better compared with nicoboxil (*p* < 0.01) and placebo (*p* < 0.0001) on all four treatment days and compared with nonivamide on Day 1 (*p* = 0.0435; Table 4). On the first treatment day, only 21.8% of nicoboxil/nonivamide patients reported ‘no’ or ‘poor’ improvement of low back mobility, compared with 63.2% for placebo, 44.8% for nicoboxil and 27.8% for nonivamide. On Day 2–4, the mobility score was still different in favour of the combination compared with placebo and nicoboxil (*p* ≤ 0.0025) but not for nonivamide (*p* ≥ 0.2289).

**Table 4:** Assessment of mobility score on Day 1 and patient assessment of efficacy on the last individual treatment day (FAS)

	Placebo	Nicoboxil	Nonivamide	Nicoboxil/Nonivamide
	*N* (%)	*N* (%)	*N* (%)	*N* (%)
Patient assessment of improvement of mobility on Day 1 (FAS)				
Number of patients *N* (%)	204 (100.0)	201 (100.0)	198 (100.0)	202 (100.0)
Improvement				
None/poor	129 (63.2)	90 (44.8)	55 (27.8)	44 (21.8)
Fair	53 (26.0)	75 (37.3)	73 (36.9)	71 (35.1)
Good	15 (7.4)	31 (15.4)	55 (27.8)	66 (32.7)
Very good	3 (1.5)	2 (1.0)	12 (6.1)	18 (8.9)
Missing	4 (2.0)	3 (1.5)	3 (1.5)	3 (1.5)
Comparison versus Nicoboxil/Nonivamide				
*p*‐value^a^	<0.0001	<0.0001	0.0435	
Odds ratio^b^ (95% CI)	6.95 (4.70, 10.28)	3.29 (2.30, 4.71)	1.43 (1.01, 2.02)	
Patient assessment of efficacy on the last individual treatment day (FAS)				
Number of patients (%)	204 (100.0)	201 (100.0)	198 (100.0)	202 (100.0)
Patient efficacy assessment				
Very good	9 (4.4)	19 (9.5)	34 (17.2)	50 (24.8)
Good	47 (23.0)	67 (33.3)	85 (42.9)	88 (43.6)
Fair	22 (10.8)	20 (10.0)	27 (13.6)	20 (9.9)
Poor	125 (61.3)	94 (46.8)	52 (26.3)	42 (20.8)
Missing	1 (0.5)	1 (0.5)	0 (0.0)	2 (1.0)
Comparison versus nicoboxil/nonivamide				
*p*‐value^a^	<0.0001	<0.0001	0.0129	
Odds ratio^b^ (95% CI)	7.38 (4.94, 11.03)	3.62 (2.47, 5.31)	1.59 (1.10, 2.30)	

a Logistic regression.

b Odds ratios were rounded to two decimals.

Patients in the nicoboxil/nonivamide group assessed the efficacy on their last individual treatment day as better than patients in the three other treatment groups (*p* ≤ 0.0129; Table 4).

The proportion of patients who assessed efficacy on their last individual treatment day as ‘very good’ or ‘good’ was highest in the nicoboxil/nonivamide group (~68%), followed by the nonivamide group (~60%), the nicoboxil group (~43%) and the placebo group (~27%). The investigator assessment of efficacy provided comparable results (data not shown).

The proportion of patients taking rescue medication was numerically highest in the placebo group (25.5%), followed by the nicoboxil group (22.4%), the nicoboxil/nonivamide group (18.3%) and the nonivamide group (17.7%).

### 3.4 Safety

A total of 66 patients experienced AEs during the study (28 patients treated with nicoboxil/nonivamide, 18 patients treated with nonivamide, 11 patients treated with nicoboxil and 9 patients treated with placebo). One SAE, which was not treatment‐related, was reported in the study: a patient in the nonivamide group was hospitalized due to severe back pain. AEs reported by 38 patients were assessed as drug‐related (Table 5).

**Table 5:** Frequency of patients with drug‐related AEs

	Placebo	Nicoboxil	Nonivamide	Nicoboxil/nonivamide	Total
	*n* (%)	*n* (%)	*n* (%)	*n* (%)	*n* (%)
Number of patients	204 (100.0)	201 (100.0)	198 (100.0)	202 (100.0)	805 (100.0)
Total with related AEs	1 (0.5)	8 (4.0)	11 (5.6)	18 (8.9)	38 (4.7)
General disorders and administration site conditions	0 (0.0)	3 (1.5)	7 (3.5)	8 (4.0)	18 (2.2)
Feeling hot	0 (0.0)	1 (0.5)	5 (2.5)	7 (3.5)	13 (1.6)
Pain	0 (0.0)	0 (0.0)	2 (1.0)	2 (1.0)	4 (0.5)
Application site burn	0 (0.0)	0 (0.0)	1 (0.5)	0 (0.0)	1 (0.1)
Application site pruritus	0 (0.0)	1 (0.5)	0 (0.0)	0 (0.0)	1 (0.1)
Hyperthermia	0 (0.0)	1 (0.5)	0 (0.0)	0 (0.0)	1 (0.1)
Local swelling	0 (0.0)	0 (0.0)	0 (0.0)	1 (0.5)	1 (0.1)
Temperature intolerance	0 (0.0)	0 (0.0)	1 (0.5)	0 (0.0)	1 (0.1)
Skin and subcutaneous tissue disorders	1 (0.5)	4 (2.0)	2 (1.0)	9 (4.5)	16 (2.0)
Erythema	0 (0.0)	1 (0.5)	1 (0.5)	6 (3.0)	8 (1.0)
Pruritus	1 (0.5)	3 (1.5)	1 (0.5)	2 (1.0)	7 (0.9)
Pain of skin	0 (0.0)	0 (0.0)	0 (0.0)	1 (0.5)	1 (0.1)
Rash	0 (0.0)	0 (0.0)	0 (0.0)	1 (0.5)	1 (0.1)
Nervous system disorders	0 (0.0)	2 (1.0)	2 (1.0)	5 (2.5)	9 (1.1)
Burning sensation	0 (0.0)	2 (1.0)	2 (1.0)	5 (2.5)	9 (1.1)
Musculoskeletal and connective tissue disorders	0 (0.0)	0 (0.0)	1 (0.5)	0 (0.0)	1 (0.1)
Back pain	0 (0.0)	0 (0.0)	1 (0.5)	0 (0.0)	1 (0.1)
Psychiatric disorders	0 (0.0)	0 (0.0)	0 (0.0)	1 (0.5)	1 (0.1)
Insomnia	0 (0.0)	0 (0.0)	0 (0.0)	1 (0.5)	1 (0.1)
Vascular disorders	0 (0.0)	1 (0.5)	0 (0.0)	0 (0.0)	1 (0.1)
Flushing	0 (0.0)	1 (0.5)	0 (0.0)	0 (0.0)	1 (0.1)

The most frequently reported drug‐related AEs on the preferred term (PT) level were feeling hot, burning sensation and erythema, which were all reported with the highest frequency in the nicoboxil/nonivamide group, followed by the nonivamide and nicoboxil groups, and did not occur in the placebo group. Pruritus was most frequently reported in the nicoboxil group. None of the drug‐related AEs was considered to be serious; all patients recovered from all drug‐related AEs.

Across all treatment groups, patients and investigators assessed the tolerability of study medication as ‘very good’ or ‘good’ for the majority of patients (data for patients not shown). Investigators assessed the final overall tolerability for the placebo (*p* < 0.0001) and nicoboxil (*p* < 0.0001) groups as better compared with the nicoboxil/nonivamide group, and for the nonivamide group (*p* = 0.7439) as similar compared with the combination group (Supporting Information Table S2).

## 4 Discussion

Nicoboxil/nonivamide ointment has been used since the 1950s to treat complaints of the musculoskeletal system. Its effects on acute low back pain, however, had not been investigated up to now in a randomized controlled trial. It has been shown that both ingredients contribute to cutaneous hyperaemia (Stuecker et al., 1999) and that dermal application of nicoboxil/nonivamide cream enhances blood supply in skin and musculature (Warnecke et al., 2013).

This large, double‐blind, randomized, parallel‐group, active‐ and placebo‐controlled, four‐arm trial showed that the non‐prescription fixed combination of nicoboxil and nonivamide effectively reduced acute non‐specific low back pain. Pain reduction developed fast and ran more or less parallel for all treatment groups from Day 1 to Day 4 (see Fig. 2), indicating fast onset of action of nicoboxil/nonivamide. Possibly treatment for more than 4 days would have induced stronger discrimination between treatment groups.

PID changes versus baseline (Table 3) have been discussed based on criteria established by Moore and colleagues (Moore et al., 2011). Reductions of acute pain intensity by at least 15% are now considered to represent a ‘minimum clinical benefit’, reductions by at least 30% correspond to a ‘moderate clinical benefit’ and reductions by at least 50% represent a ‘substantial clinical benefit’. Thus, nicoboxil/nonivamide provided minimum clinical benefit after 4 h, moderate benefit after 8 h, and substantial benefit at the last individual treatment day, whereas placebo yielded only minimum clinical benefit or below during the treatment period. To get an impression of functional recovery, patients were asked to assess their low back mobility on each treatment day. On the first treatment day, only 8.9% of patients in the placebo group reported ‘very good’ or ‘good’ improvement, compared with 41.6% in the nicoboxil/nonivamide group. Thus, the combination provided not only robust pain relief but also early functional recovery in a high proportion of patients. Together with the pronounced pain relief this could explain the high percentage of patients assessing the efficacy as ‘very good’ or ‘good’.

In contrast to the high burden of (acute) non‐specific low back pain, clinically proven treatment options are limited. Early intervention is desirable to get patients back to their normal activities as soon as possible and to prevent chronification. A recent review of the current (inter‐) national clinical guidelines for the management of low back pain mentions early return to work (despite having low back pain) in their list of recommendations. Paracetamol is usually recommended as a first‐line therapy and NSAIDs (non‐steroidal anti‐inflammatory drugs) as second‐line therapy when paracetamol is not sufficient (Koes et al., 2010). Because paracetamol is recommended as first‐line treatment option by many guidelines, and has been used as rescue medication in other trials [e.g. investigating NSAIDs (Dreiser et al., 2003) or opioids (Buynak et al., 2010)], this drug was chosen as rescue medication also for our trial. Despite these recommendations, data on the effects of these drugs for treating acute low back pain are sparse and the use is limited by their safety/tolerability profile and restrictions in certain patient groups. Although paracetamol is recommended as first‐line pharmacological treatment by the majority of guidelines, a recent clinical trial showed that this analgesic was not superior to placebo (Williams et al., 2014). Having this information in mind, paracetamol can be considered not to be the appropriate rescue medication for future clinical trials investigating acute low back pain. A Cochrane review (Roelofs et al., 2008) identified only seven high‐quality studies comparing NSAIDs with placebo for the treatment of acute low back pain. This is consistent with a meta‐analysis performed by Keller and colleagues (Keller et al., 2007), in which the effect sizes of treatments for non‐specific low back pain in controlled randomized trials were compared with groups without active treatment. The authors calculated standardized mean differences (SMDs), i.e. the differences in outcome measures between two treatment groups divided by the pooled standard deviation. SMDs of 0.2–0.5 were considered as ‘small’, of 0.5–0.8 as ‘moderate’ and of >0.8 as ‘large’. The authors concluded that SMDs for effect sizes of orally administered non‐steroidal anti‐inflammatory drugs (prescription, as well as non‐prescription analgesics) were only modest for acute back pain (SMD of 0.51); the effect sizes found for other treatment options were even smaller. It was concluded that there is an urgent need for developing more effective interventions. Based on the calculation method described (Keller et al., 2007), a SMD of 0.778 for the effect size of nicoboxil/nonivamide ointment versus placebo in terms of the primary endpoint was calculated. Thus, the treatment effect of nicoboxil/nonivamide can be considered as almost ‘large’. The corresponding value for over‐the‐counter NSAIDs was calculated to be 0.41 only [referring to a study by Dreiser et al., comparing the effects of ibuprofen, diclofenac and placebo (Dreiser et al., 2003)]. A larger effect size was only achieved by injections of diclofenac and metamizole [SMD = 0.84 (Babej‐Doelle et al., 1994)], i.e. with interventions that are much more interfering and invasive compared with topical treatment.

For other treatment options such as prescription‐only muscle relaxants (cyclobenzapride, tizanidine or thiocolchicoside), the pooled effect size was 0.52, lower than the SMD for nicoboxil/nonivamide ointment (Keller et al., 2007). It has to be taken into account, that comparisons over different studies bear some limitations since the conditions of the analysed studies might differ (e. g. due to the route of administration – topical treatments might be perceived differently from systemic treatments, especially when they induce perceptible effects like hyperaemia). However, the analysis of SMDs provides a basis for comparing such data.

Tolerability of the treatments in this study was generally good. Nicoboxil/nonivamide had the highest rate of AEs, which were all expected and related to the mechanism of action. As this ointment was developed with the intention of generating strong heat sensation and cutaneous hyperaemia, the reporting of such sensations as AEs by some patients is not surprising.

Limitations of this study include that superiority of the combination over nonivamide alone could not be shown concerning the primary endpoint. However, it has to be considered that ointments with the individual ingredients at these doses are not commercially available, and that the combination was shown to be superior to all other treatment arms for several clinically relevant endpoints, such as APID on the last individual treatment day, mobility score on the first treatment day and patient assessment of efficacy. The overall AE frequency in this study was low, and drug‐related AEs were consistent with the mechanism of action of hyperaemia‐inducing agents. Even most of the 18 patients in the nicoboxil/nonivamide arm who reported AEs experienced pain relief (i.e. benefited) from the treatment (data not shown). Assessment of tolerability by patients and investigators was not different for nonivamide and nicoboxil/nonivamide (*p* > 0.4249). Thus, the combination in total provided more benefit, and comparable risk when compared with nonivamide alone.

One might argue that treatments inducing hyperaemia cannot be fully blinded. However, we think that the risk of unblinding was relatively low. In this parallel‐group trial, every patient experienced only one treatment, and therefore comparison with other treatments was not possible. It is known that the individual response to the local effects of nicoboxil/nonivamide products vary considerably from patient to patient. Furthermore, the application of an ointment per se can induce the perception of heat due to the mechanical stimulation of the skin, as well as effects on perspiration and thermal isolation.

The maximum treatment duration of 4 days might be considered as too short compared with other studies investigating the treatment of acute low back pain, which often have duration of 7 days. However, based on the results of this study, the maximum possible effect of nicoboxil/nonivamide ointment might have been even pronounced after 7 days of treatment.

In summary, this study demonstrated that nicoboxil/nonivamide ointment is an effective, tolerable, and safe treatment option for acute low back pain. Based on published data by others, the efficacy seems to be comparable or superior to that of systemic over‐the‐counter, as well as some prescription analgesics and muscle relaxants, while – as a topical treatment – has a low risk of systemic side effects, so that this combination adds a promising option for the treatment of acute low back pain.

## Author contributions

M.G. and T.S. substantially contributed to study concept, design and results interpretation; C.H., E.R., W.P.‐R., T.W. substantially contributed to study concept, design, analysis, results interpretation and drafted the manuscript. All authors critically revised the manuscript for important intellectual content and approved it.

## Study registration numbers

EudraCT No.: 2011‐003890‐27; Clinical Trials.gov identifier: NCT01708915

## Supporting information


**Figure S1**. Patient disposition. All enrolled patients were randomized, treated and provided data for the primary endpoint. FAS = full analysis set; IPV = important protocol violation; PPS = per‐protocol set. Patients may have had more than one important protocol violation. Only IPV categories with at least one IPV are presented.

**Table S1.** Frequency distribution, Kaplan–Meier analysis and log rank test of the time to onset of pain relief after the first application.

**Table S2.** Final overall investigator assessment of tolera‐bility – TS.
