# Supplementary material for: Efficacy and safety of nicoboxil/nonivamide ointment for the treatment of acute pain in the low back – A randomized, controlled trial
Source: Eur J Pain. 2015 Apr 30;20(2):263–73. doi: 10.1002/ejp.719 (PMC5029595; doi:10.1002/ejp.719)
Supplement: Supplementary file 1 — Figure S1. Patient disposition. All enrolled patients were randomized, treated and provided data for the primary endpoint. FAS = full analysis set; IPV = important protocol violation; PPS = per‐protocol set. Patients may have had more than one important protocol violation. Only IPV categories with at least one IPV are presented. [file EJP-20-263-s001.pdf]

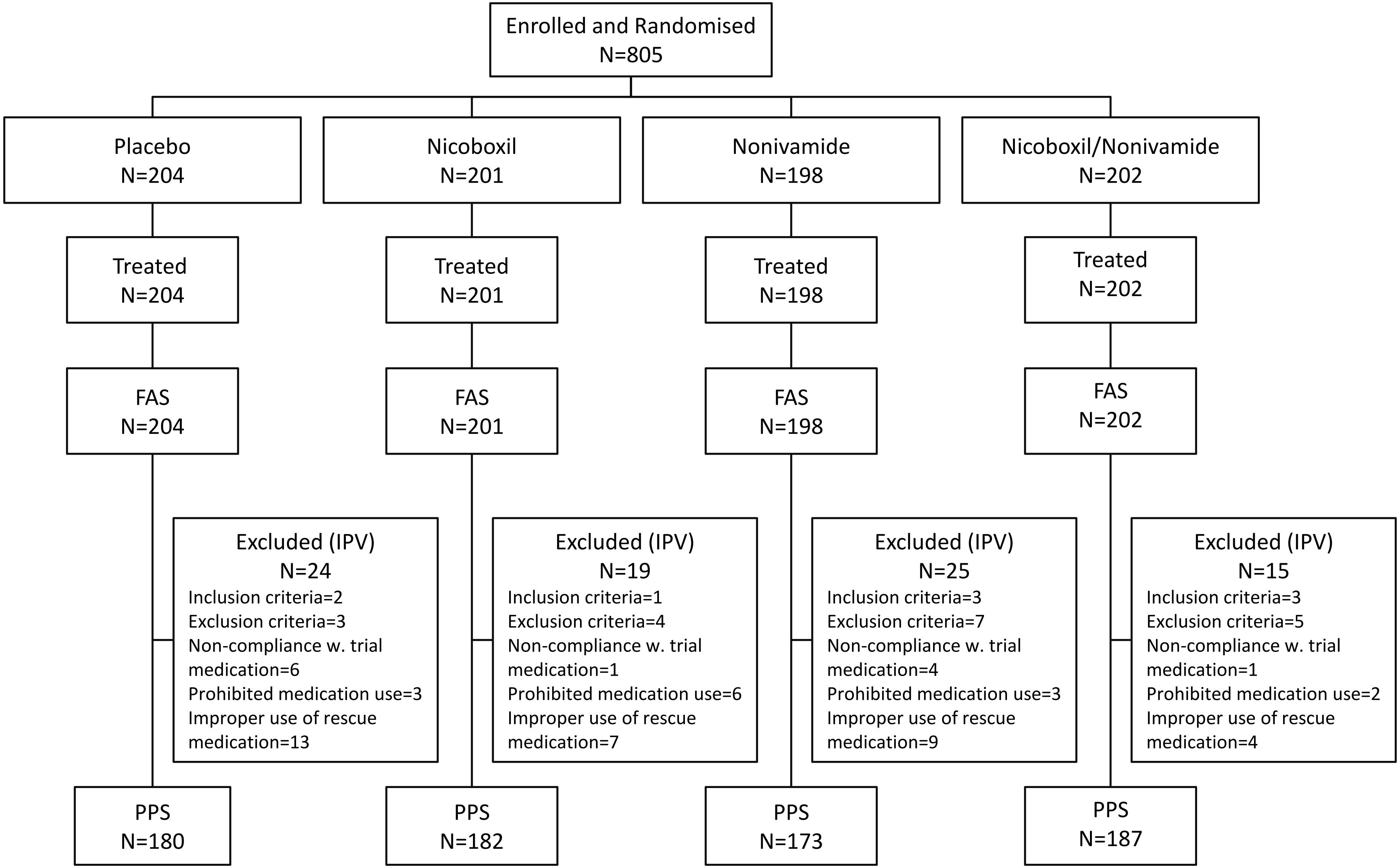

Patient disposition. All enrolled patients were randomised, treated, and provided data for the primary endpoint. FAS = full analysis set, IPV = important protocol violation, PPS = per protocol set. Patients may have had more than one important protocol violation. Only IPV categories with at least one IPV are presented.
