# Supplementary material for: Efficacy and safety of nicoboxil/nonivamide ointment for the treatment of acute pain in the low back – A randomized, controlled trial
Source: Eur J Pain. 2015 Apr 30;20(2):263–73. doi: 10.1002/ejp.719 (PMC5029595; doi:10.1002/ejp.719)
Supplement: Supplementary file 2 — Table S1. Frequency distribution, Kaplan–Meier analysis and log rank test of the time to onset of pain relief after the first application. [file EJP-20-263-s002.doc]

**Table S1**

Frequency distribution, Kaplan-Meier analysis, and log rank test of the time to onset of pain relief after the first application

|  | **Placebo** | **Nicoboxil** | **Nonivamide** | **Nixoboxil/nonivamide** |
| --- | --- | --- | --- | --- |
| **Frequency distribution** |  |  |  |  |
| Number of patients with available data, n (%) | 201 (100.00) | 198 (100.0) | 198 (100.0) | 200 (100.0) |
| **Time to onset of pain relief, n (%)** |  |  |  |  |
| Within 30 min | 12 (6.0) | 18 (9.1) | 32 (16.2) | 34 (17.0) |
| Between 30 min and 1 h | 17 (8.5) | 32 (16.2) | 28 (14.1) | 45 (22.5) |
| Between 1 and 2 h | 17 (8.5) | 25 (12.6) | 39 (19.7) | 31 (15.5) |
| Between 2 and 4 h | 15 (7.5) | 21 (10.6) | 28 (14.1) | 32 (16.0) |
| Between 4 and 8 h | 33 (16.4) | 31 (15.7) | 36 (18.2) | 25 (12.5) |
| After more than 8 h | 8 (4.0) | 6 (3.0) | 11 (5.6) | 7 (3.5) |
| No effect | 99 (49.3) | 65 (32.8) | 24 (12.1) | 26 (13.0) |
| **Kaplan-Meier analysis and log rank test** |  |  |  |  |
| Median category | 5  (after more than 8 h) | 4  (between 4 and 8 h) | 3  (between 2 and 4 h) | 2  (between 1 and 2 h) |
| **Comparison versus nicoboxil/nonivamide** |  |  |  |  |
| p-value^a^ | < 0.0001 | < 0.0001 | 0.3828 | - |

^a^Log-rank test stratifying for baseline PI
