# Supplementary material for: Efficacy and safety of nicoboxil/nonivamide ointment for the treatment of acute pain in the low back – A randomized, controlled trial
Source: Eur J Pain. 2015 Apr 30;20(2):263–73. doi: 10.1002/ejp.719 (PMC5029595; doi:10.1002/ejp.719)
Supplement: Supplementary file 3 — Table S2. Final overall investigator assessment of tolera‐bility – TS. [file EJP-20-263-s003.doc]

**Table S2**

Final overall investigator assessment of tolerability - TS

|  | **Placebo** | **Nicoboxil** | **Nonivamide** | **Nicoboxil/**  **Nonivamide** |
| --- | --- | --- | --- | --- |
|  | **N (%)** | **N (%)** | **N (%)** | **N (%)** |
| **Number of patients** | 204 (100.0) | 201 (100.0) | 198 (100.0) | 202 (100.0) |
| **Investigator assessment of**  **tolerability** | | | | |
| Very good | 115 (56.4) | 104 (51.7) | 60 (30.3) | 66 (32.7) |
| Good | 86 (42.2) | 86 (42.8) | 108 (54.5) | 94 (46.5) |
| Fair | 2 (1.0) | 8 (4.0) | 18 (9.1) | 28 (13.9) |
| Poor | 1 (0.5) | 2 (1.0) | 12 (6.1) | 13 (6.4) |
| Missing | 0 (0.0) | 1 (0.5) | 0 (0.0) | 1 (0.5) |
| **Comparison versus**  **nicoboxil/nonivamide** | | | | |
| p-value^a^ | < 0.0001 | < 0.0001 | 0.7439 |  |
| Odds ratio^b^ (95% CIs) | 0.25 (0.17, 0.39) | 0.33 (0.22, 0.50) | 1.07 (0.72, 1.60) |  |

^a^The logistic regression model included baseline pain intensity, centre, and treatment.

^b^Odds ratios were rounded to two decimals.
